# Supplementary material for: Vitellogenins Level as a Biomarker of the Honeybee Colony Strength in Urban and Rural Conditions
Source: Insects. 2024 Dec 29;16(1):25. doi: 10.3390/insects16010025 (PMC11766442; doi:10.3390/insects16010025)

Fig. S1. Details of the experimental set-ups: (A) two apiaries, six honey bee (*Apis mellifera*) colonies for each apiary with queen bees – sisters from pure breeding lines, artificially inseminated with semen from the same pool of drones, (B) Bee colony strength and the vitellogenin level measurements in the brain, fat body, and the entire body of bees from May (V) to August (VIII). Created in BioRender. Nicewicz, A. (2024) https://BioRender.com/o74y870.

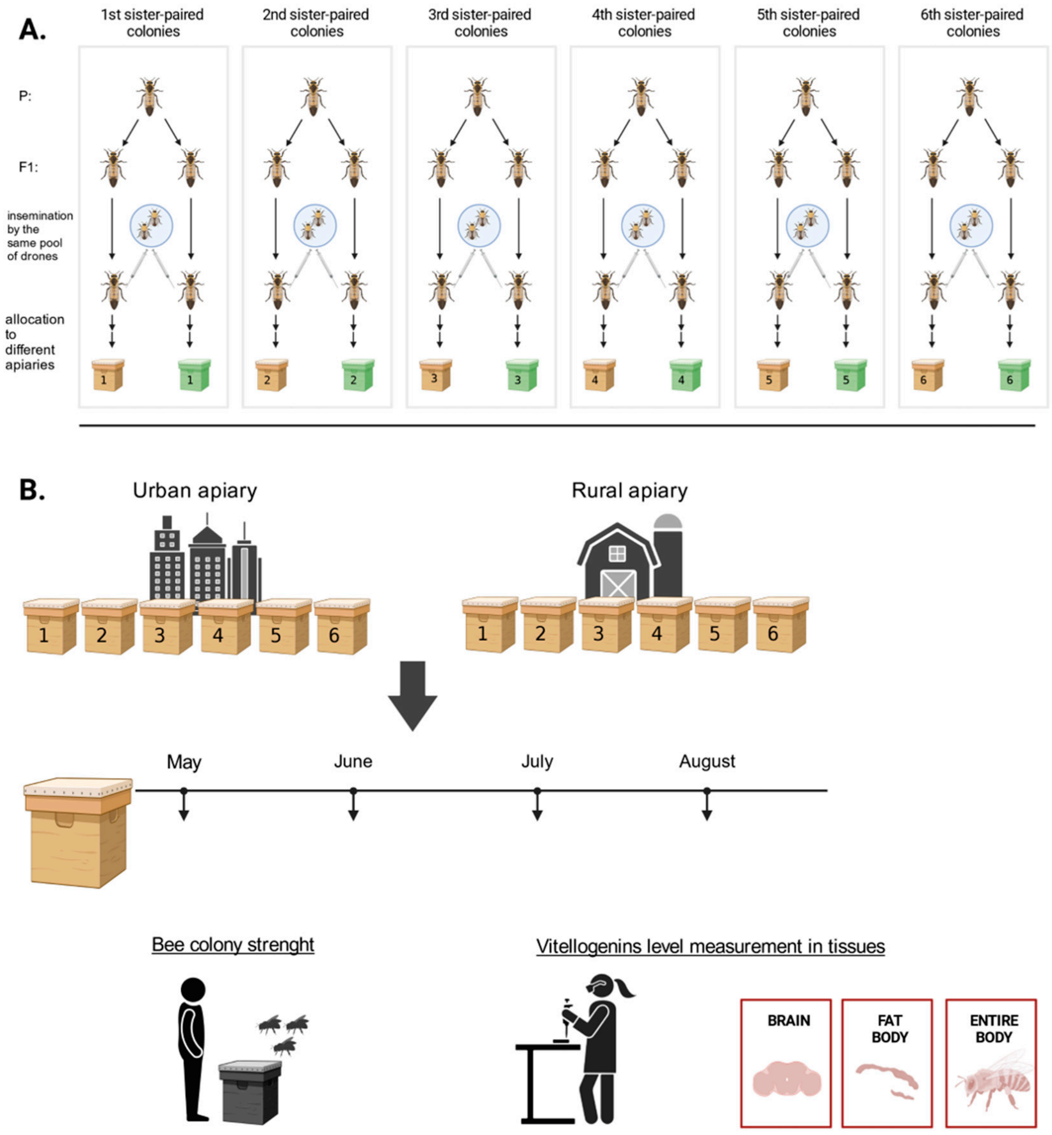

Table S1. Heat map of the statistically significant differences between the numbers of individuals in bee colonies (1-6) within one apiary (U – urban or R – rural) from May (V) to August (VIII). Sidak's multiple comparison tests, $p \leq 0.05$.

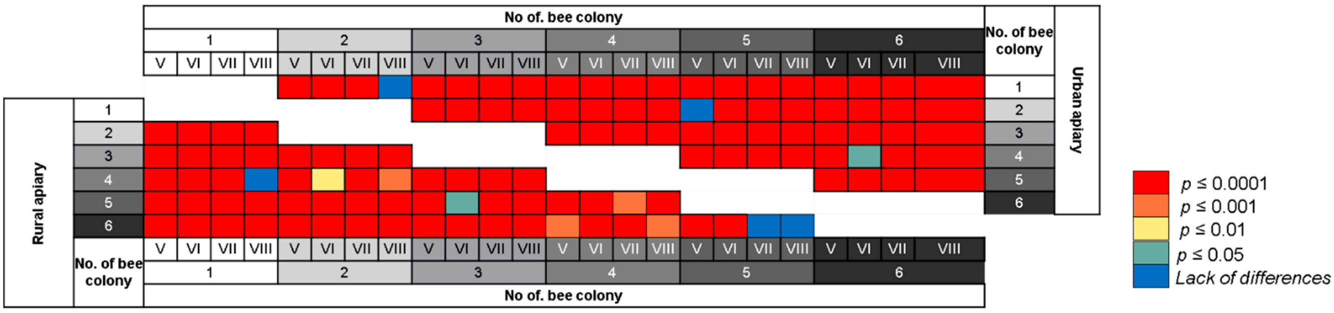

Table S2. Heat map of the statistically significant differences between the concentration of vitellogenins in the entire body (A), brain (B) and fat body (C) of *Apis mellifera carnica* foragers from colonies (1-6) within the same apiary (U – urban or R – rural) collected in the months from May (V) to August (VIII). Sidak's multiple comparison tests, $p \leq 0.05$.

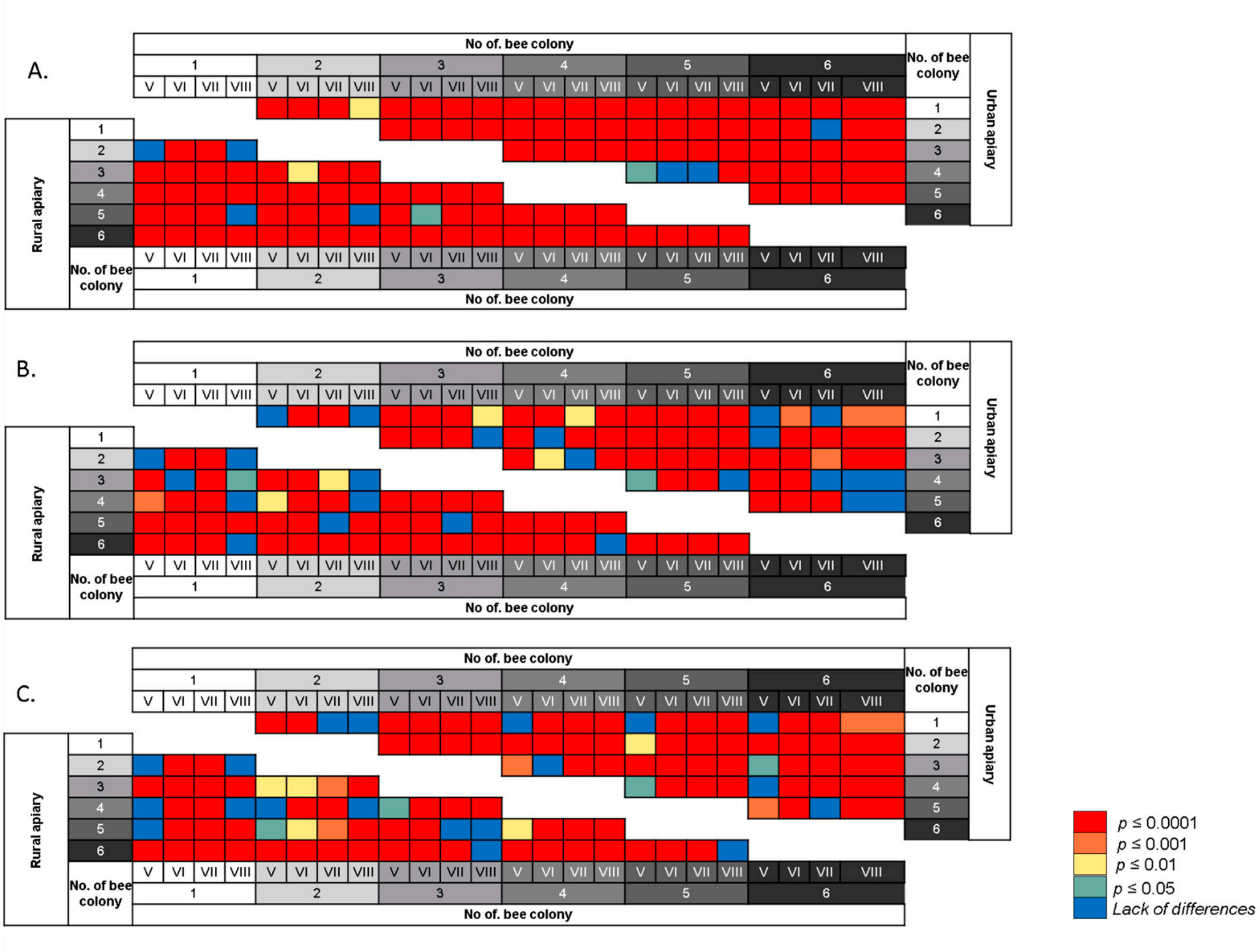

Supplement: Supplementary file 1 [file insects-16-00025-s001.zip › insects-3343153-supplementary.pdf]
